# PRDM4 inhibits cell proliferation and tumorigenesis by inactivating the PI3K/AKT signaling pathway through targeting of PTEN in cervical carcinoma

**DOI:** 10.1038/s41388-021-01765-x

**Published:** 2021-04-12

**Authors:** Wen-Ting Yang, Mei Chen, Rui Xu, Peng-Sheng Zheng

**Affiliations:** 1 Department of Reproductive Medicine, The First Affiliated Hospital of Xi’an Jiaotong University, Xi’an, Shaanxi People’s Republic of China; 2 Key Laboratory of Environment and Genes Related to Diseases, Ministry of Education of the People’s Republic of China, Xi’an, Shaanxi People’s Republic of China; 3 Department of Internal Medicine One, Shaanxi Cancer Hospital, College of Medicine, Xi’an Jiaotong University, Xi’an, People’s Republic of China

**Keywords:** Cervical cancer, Cell growth

## Abstract

PR domain zinc finger protein 4 (PRDM4) is a transcription factor that plays key roles in stem cell self-renewal and tumorigenesis. However, its biological role and exact mechanism in cervical cancer remain unknown. Here, both immunohistochemistry (IHC) and Western blot assays demonstrated that the expression of PRDM4 in cervical cancer tissues was much lower than that in the normal cervix. A xenograft assay showed that PRDM4 overexpression in the cervical cancer cell lines SiHa and HeLa dramatically inhibited cell proliferation and tumorigenic potential in vivo. Conversely, the silencing of PRDM4 promoted cervical cancer cell proliferation and tumorigenic potential. Mechanistically, PRDM4 induced cell cycle arrest at the transition from G0/G1 phase to S phase by upregulating p27 and p21 expression and downregulating Cyclin D1 and CDK4 expression. Furthermore, the PI3K/AKT signaling pathway was inactivated in PRDM4-overexpressing cells, which decreased the levels of p-AKT and upregulated the expression of PTEN, an inhibitor of the PI3K/AKT signaling pathway, at both the transcriptional and translational levels. Dual-luciferase reporter assays and qChIP assays confirmed that PRDM4 transactivated the expression of PTEN by binding to two specific regions in the *PTEN* promoter. Furthermore, PTEN silencing or a PTEN inhibitor rescued the cell defects induced by PRDM4 overexpression. Therefore, our data suggest that PRDM4 inhibits cell proliferation and tumorigenesis by downregulating the activity of the PI3K/AKT signaling pathway by directly transactivating PTEN expression in cervical cancer.

## Introduction

Cervical cancer is the fourth most commonly diagnosed cancer worldwide and is still the second leading cause of cancer-related death among women in developing countries [1]. Epidemiological case series have shown that 99.7% of patients with cervical carcinoma are positive for human papillomavirus (HPV) [2]. HPV16 and HPV18 are high-risk carcinogenic HPV genotypes that account for ~55–60% and 10–15% of all cervical cancers, respectively [2–4]. However, the activation of oncogenes and the inactivation of tumor suppressors are also necessary for tumorigenesis and the development of cervical cancer. For example, TP53 protein overexpression during cervical cancer tumorigenesis could play a pivotal role in cervical cancer progression as a late event [5]. In recent years, epigenetic mechanisms, including methylation and noncoding RNA, have also been shown to regulate the progression of cervical cancer [6]. Additionally, promoter methylation and the loss of the expression of PTEN, which acts as an antioncogene, occur frequently in carcinomas of the uterine cervix [7]. Previously, our laboratory clarified that some stem cell-related genes are associated with cervical carcinogenesis. For example, SOX17 and DAX1 can promote Wnt/β-catenin signaling-dependent cell expansion in cervical carcinoma [8, 9]. Moreover, SLUG, SOX9, and KLF4 have been demonstrated to suppress cervical cancer cell growth in vivo by different mechanisms [10–12].

PRDM family proteins are characterized by the presence of highly conserved structural domains, the so-called positive regulatory/suppressor of variegation, enhancer of zeste, trithorax and C_2_H_2_-like zinc finger domains [13]. As transcriptional regulators, these molecules often control various aspects of cell differentiation and organism development, and when dysregulated, they are involved in carcinogenesis [14–17]. Recent reports have suggested that PRDM4 is implicated in cell signal transduction, cell proliferation, and differentiation [18, 19]. Furthermore, the loss of PRDM4 was associated with poor prognosis in patients with gastric cancer and may act as a tumor suppressor [20]. However, PRDM4 promotes cell invasion and metastasis mediated by the Hippo signaling pathway by activating integrin β2 in pancreatic cancer [21], suggesting a heterogeneous role in different tissue types. To the best of our knowledge, there have been no reports on the function of PRDM4 in cervical carcinoma.

At present, we are the first to reveal that PRDM4 could inhibit cell proliferation and tumor formation in cervical cancer by inactivating the PI3K/AKT signaling pathway by transactivating the expression of the suppressor PTEN.

## Results

### Expression of the PRDM4 protein in normal cervix and different cervical lesions

**Fig. 1:**
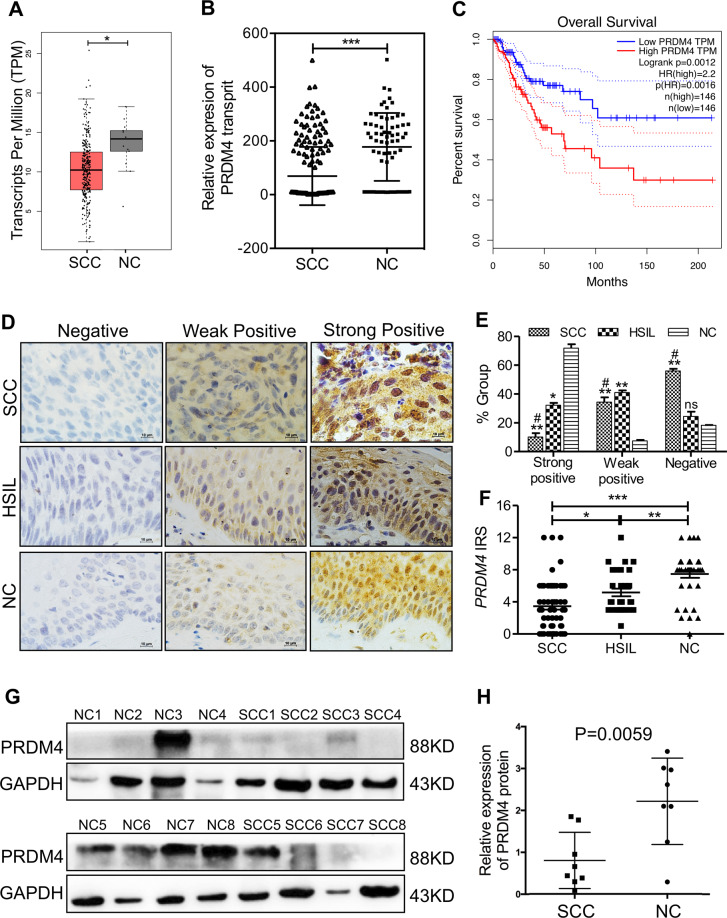
Expression of PRDM4 protein in normal cervix and different cervical lesions. **A** The dataset from the GEPIA (Gene Expression Profiling Interactive Analysis) repository (http://gepia.cancer-pku.cn/) showed the *PRDM4* mRNA levels in 304 cervical cancer tissues and 12 normal cervical tissues. **B** The Gene Expression Omnibus (GEO) database showed higher *PRDM4* mRNA levels in 197 cervical cancer tissues than in 68 normal cervical tissues. **C** Survival analyses were performed with the Kaplan–Meier estimator from the Kaplan–Meier plotter based on the TCGA database. **D** Immunohistochemical staining for PRDM4 protein in 30 NC, 38 HSIL, and 60 SCC specimens (scale bar: 10 µm). **E** The percentages of negative, weakly positive, and strongly positive PRDM4 expression are shown. **F** Semiquantitative analysis of the IRS of PRDM4 expression is illustrated. **G**, **H** The expression of PRDM4 protein was detected by Western blots in eight primary SCC and eight NC specimens, and semiquantitative analysis by comparison to GAPDH was performed. Error bars represent SD. *p < 0.05.

To explore the role of PRDM4 in the development and progression of cervical cancer, we examined the mRNA levels of *PRDM4* in 304 patients with cervical cancer and found that it was significantly lower than that in 13 patients without malignant tumors in The Cancer Genome Atlas (TCGA) RNA-Seq database (Fig. 1A, *p* < 0.05). Additionally, the Gene Expression Omnibus database showed a much lower *PRDM4* mRNA level in 197 cervical cancer tissues than in 68 normal cervical tissues (Fig. 1B, *p* < 0.05). Moreover, as the *PRDM4* expression level increased, the probability of cervical cancer patient survival significantly increased, as shown by the Kaplan–Meier estimator (Fig. 1C, *p* = 0.0012).

In our clinical specimens, PRDM4 protein expression was detected in normal cervix (NC, *N* = 38), high-grade squamous intraepithelial lesions (HSILs, *N* = 30), and invasive cervical squamous cell carcinoma (SCC, *N* = 60) using immunohistochemistry (IHC, Fig. 1D). The percentage of negative PRDM4 staining was 53.33% in SCC (32/60), 26.67% in HSILs (8/30), and 18.42% in NC (7/38). The positive staining rate (including weak positive and strong positive) of the PRDM4 protein was significantly higher in NC than in SCC (71.58 vs 46.67%, Fig. 1E, *p* < 0.01). Moreover, the immunoreactivity score (IRS) of PRDM4 staining decreased from 7.46 ± 0.48 in NC to 6.33 ± 0.438 in HSILs and then to 3.45 ± 0.40 in SCC (Fig. 1F, *p* < 0.01). Statistical evaluation of the immunohistochemical staining showed that the expression level of PRDM4 was significantly associated with clinical stage and LN metastasis (*p* < 0.05) but was not associated with age, tumor size, or histological grade (Table 1).

**Table 1:** Relationship between PRDM4 protein and the characteristics of cervical cancers.

Characteristics	*n*	PRDM4 expression		*p* value
		Positive	Negative	
Age, years				
≤45	26	10	16	0.3052
>45	34	18	16	
Clinical stage				
I–II	42	27	25	0.0005*
III–IV	18	1	17	
SCC differentiation				
Well	15	8	7	0.5672
Moderate–poor	45	20	25	
Lymph node metastasis				
Absence	48	26	22	0.0253*
Present	12	2	10	

For comparison among groups, the *χ*^2^ test was performed. A *p* value <0.05 was considered statistically significant.

*p < 0.05.

Additionally, the PRDM4 protein was detected by Western blots in eight NC and eight SCC tissues, all of which were selected randomly (Fig. 1G), and the relative expression of PRDM4 to GAPDH was much higher in the NC than in SCC tissues (Fig. 1H, *p* = 0.0059). These results suggested that the PRDM4 protein was downregulated in cervical cancer and might have a tumor suppressive function.

### PRDM4 inhibited tumor growth of cervical cancer cells in vivo

**Fig. 2:**
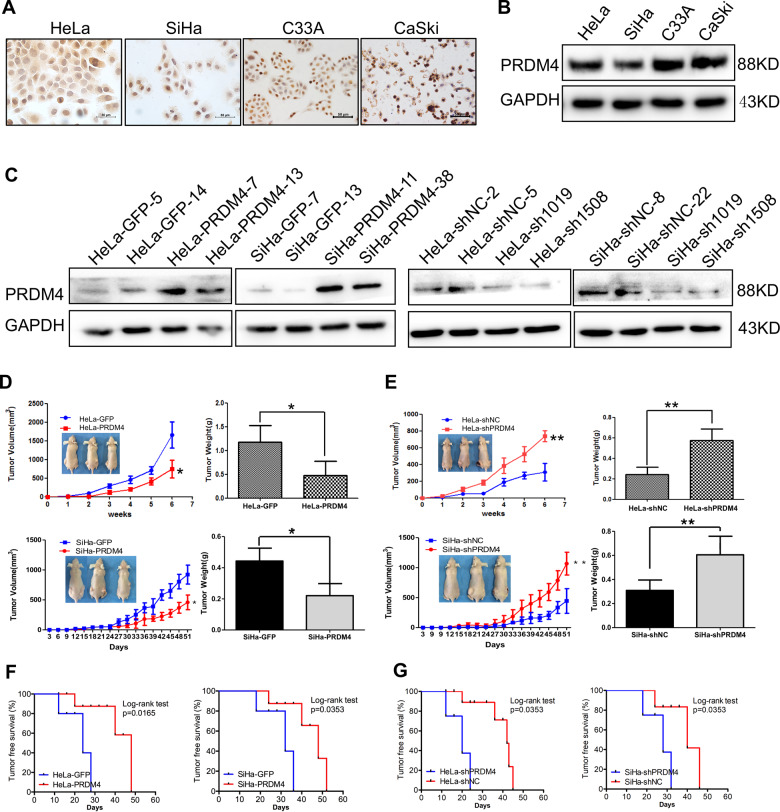
PRDM4 inhibited the tumor formation of cervical cancer cells in vivo. The expression of PRDM4 protein in cervical cancer cell lines was detected by IHC staining (**A**) and Western blots (**B**). **C** PRDM4 protein was detected in the PRDM4-overexpressing or PRDM4-silenced HeLa and SiHa cells. **D**, **E** The tumor growth curve and weight were analyzed in the PRDM4-overexpressing or PRDM4-silenced HeLa and SiHa cells with six female nude mice in triplicate. **F**, **G** Tumor-free survival was analyzed in the PRDM4-overexpressing or PRDM4-silenced HeLa and SiHa cells. Scale bar: 50 µm. *p < 0.05 and **p < 0.01.

Immunocytochemistry and Western blot assays revealed PRDM4 expression in the cervical cancer cell lines HeLa, SiHa, C33A, and CaSki (Fig. 2A, B). Next, we generated HeLa and SiHa cell lines with stable overexpression and silencing of PRDM4 using two independent targeted short hairpin RNAs (shRNAs) (Fig. 2C). For analysis of the function of PRDM4 in cervical cancer cells in vivo, a total of 1 × 10^6^ PRDM4-overexpressing HeLa or SiHa cells, as well as the respective control cells, were injected subcutaneously into female nude mice at the same time to assess the tumor formation potential. The tumors formed by the HeLa-PRDM4 and SiHa-PRDM4 cells had a twofold smaller volume than those formed by the GFP control cells (Fig. 2D left, *p* < 0.05). Additionally, the weights of the tumors derived from the PRDM4-overexpressing HeLa and SiHa cells were much lower than those from the GFP control cells (Fig. 2D right, *p* < 0.05), and these mice also exhibited longer tumor-free survival (Fig. 2F, *p* < 005). Similarly, compared to the control mice, the mice implanted with the PRDM4-silenced HeLa and SiHa cells (10^5^ cells) showed larger tumor volumes, with a 2.6–3.7-fold increase in tumor size and a 1.7–2.1-fold increase in tumor weight; these mice also had a shortened tumor-free survival (Fig. 2E, G, *p* < 0.05). All of these results demonstrated that PRDM4 suppressed the tumor growth of cervical cancer in vivo.

### PRDM4 inhibited the proliferation of cervical cancer cells in vitro by arresting the cell cycle at the G0/G1 to S phase transition

**Fig. 3:**
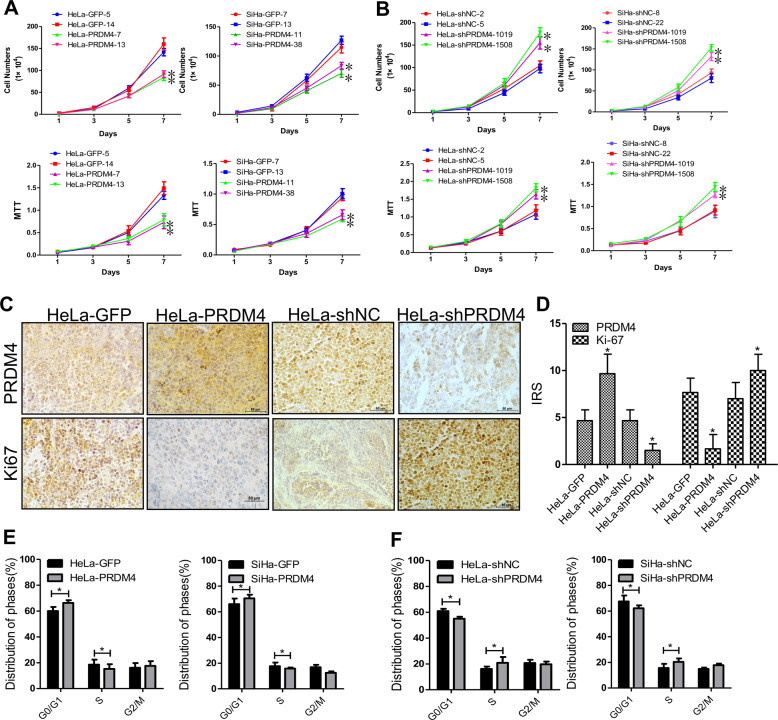
PRDM4 inhibited cell proliferation by blocking the cell cycle transition from G0/G1 to S phase. **A** Cell viability was determined in the PRDM4-overexpressing or PRDM4-silenced HeLa and SiHa cells by MTT assays. **B** Cell proliferation assays were performed in the PRDM4-overexpressing or PRDM4-silenced HeLa and SiHa cells. **C**, **D** The expression levels of PRDM4 and Ki67 in the tumor tissues are shown. **E**, **F** Cell cycle distribution was analyzed by FACS in the PRDM4-overexpressing or PRDM4-silenced cells. *p < 0.05 and **p < 0.01.

To explore the mechanism by which PRDM4 inhibited the tumor formation of cervical cancer in vivo, we evaluated whether PRDM4 overexpression or silencing could influence cell proliferation by cell counting, cell viability, and tumorsphere formation assays in vitro. The cell proliferative abilities were significantly reduced in the HeLa and SiHa cells with ectopic expression of PRDM4 compared with the HeLa-GFP and SiHa-GFP cells, respectively (Fig. 3A, *p* < 0.05). Conversely, the PRDM4-silenced HeLa and SiHa cells exhibited markedly increased cell proliferation and viability relative to those with the control shRNA (Fig. 3B). Similarly, the percentages of tumorsphere formation by the PRDM4-overexpressing HeLa and SiHa cells were 11 ± 1.4% and 13.5 ± 2.12%, which were much lower than those of the control cells at 24.5 ± 2.12% and 30 ± 2.83%, respectively (Supplementary Fig. 1A, B, *p* < 0.05). PRDM4 silencing significantly increased the tumorsphere formation in both HeLa and SiHa cells (Supplementary Fig. 1C, D, *p* < 0.05). In addition, the IHC score for Ki67 staining was significantly decreased in the tumor tissues formed by the PRDM4-overexpressing cells but dramatically increased in the tumors with stable silencing of PRDM4 compared to the control tumors (Fig. 3C, D, *p* < 0.05).

To investigate the mechanism by which PRDM4 inhibited cervical cancer cells, we obtained cell cycle profiles by FACS analysis. The upregulation of PRDM4 in HeLa and SiHa cells resulted in an increased percentage of cells in G0/G1 phase and a decreased percentage of cells in S phase compared with those of the control cells (Fig. 3E, *p* < 0.05). However, PRDM4 silencing accelerated the G1/S transition, decreasing the percentage of HeLa and SiHa cells in G0/G1 phase, and increasing the percentage of cells in S phase (Fig. 3F, *p* < 0.05). However, the apoptosis ability employed in PRDM4-mediated cervical cells showed no significant difference (Supplementary Fig. 2A, *p* > 0.05). Collectively, the cell cycle profiles suggested that PRDM4 inhibited the cell cycle transition from G1/S to S phase.

### PRDM4 inhibited cervical cancer cell proliferation by regulating cell cycle-related proteins

**Fig. 4:**
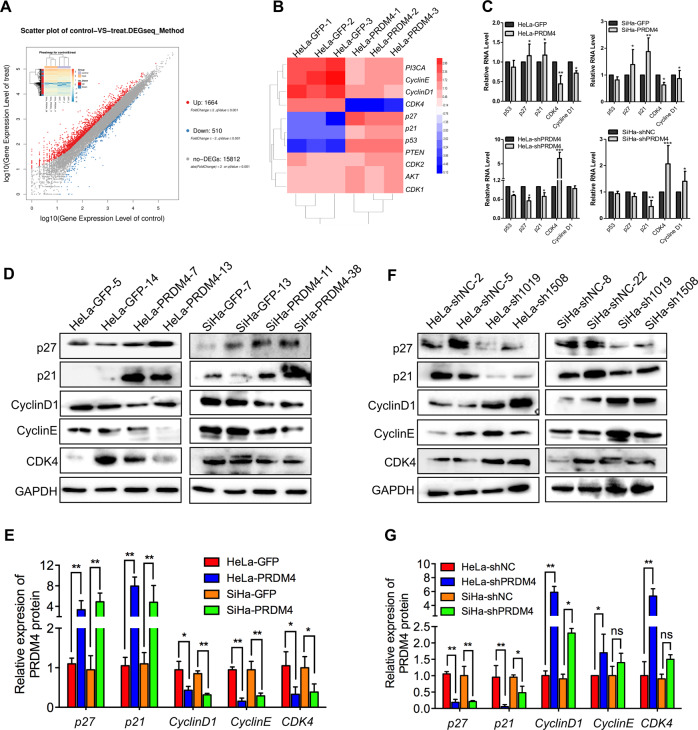
PRDM4 inhibited cell proliferation by regulating cell cycle-related proteins. **A** The differential mRNA expression in HeLa-PRDM4 and HeLa-GFP cells determined by RNA-seq showed 1664 upregulated genes and 510 downregulated genes. **B** Cell cycle-related gene levels between the HeLa-PRDM4 and HeLa-GFP cells. **C** The transcript levels of *p53*, *p21*, *p27*, *Cyclin D1*, and *CDK4* were detected in the PRDM4-overexpressing and PRDM4-silenced cells by real-time PCR. **D**–**G** Cell cycle proteins were detected by Western blotting, analyzed by utilizing a protein imprinting imaging system (Tanon 5200, China) and expressed as relative expression after normalization to GAPDH. *p < 0.05 and **p < 0.01, while ns indicates no significant difference.

To explore the molecular function of PRDM4 in cervical cancer cells, we performed a transcriptome sequencing analysis of three HeLa-PRDM4 monoclonal cell lines and HeLa-GFP monoclonal cell lines, and a total of 15,812 genes were detected. Gene Ontology enrichment analysis for all six samples revealed that 1664 gene sets were upregulated and 510 gene sets were downregulated (Fig. 4A). In these gene sets, we identified 11 genes involved in cell cycle progression that showed significant changes in expression between the two groups (Fig. 4B). For instance, the levels of the cell cycle inhibitors *p27* and *p21* were upregulated in the PRDM4-overexpressing HeLa and SiHa cells but downregulated in the PRDM4-silenced HeLa and SiHa cells, as shown by real-time PCR. However, the cell cycle promoters *CDK4* and *Cyclin D1* were decreased in the HeLa-PRDM4 and SiHa-PRDM4 cells and increased in the HeLa-shPRDM4 and SiHa-shPRDM4 cells compared with the respective control cells (Fig. 4C, *p* < 0.05). Moreover, Western blot analysis showed that the protein expression levels of p21 and p27 were higher and those of Cyclin D1 and CDK4 were much lower in the PRDM4-overexpressing cells than in the respective control cells, which was consistent with the mRNA results (*p* < 0.5, Fig. 4D, E). Furthermore, in the PRDM4-silenced HeLa and SiHa cells, the p27 and p21 proteins were increased and the Cyclin D1 and CDK4 proteins were decreased compared with those in the control cells (*p* < 0.5, Fig. 4F, G). All of these results indicated that PRDM4 inhibited cell proliferation and tumor growth, possibly by regulating cell cycle-related genes, including p27, p21, Cyclin D1, and CDK4, in cervical cancer.

### PRDM4 inhibited the activity of the PI3K/AKT signaling pathway by transactivating the expression of PTEN

**Fig. 5:**
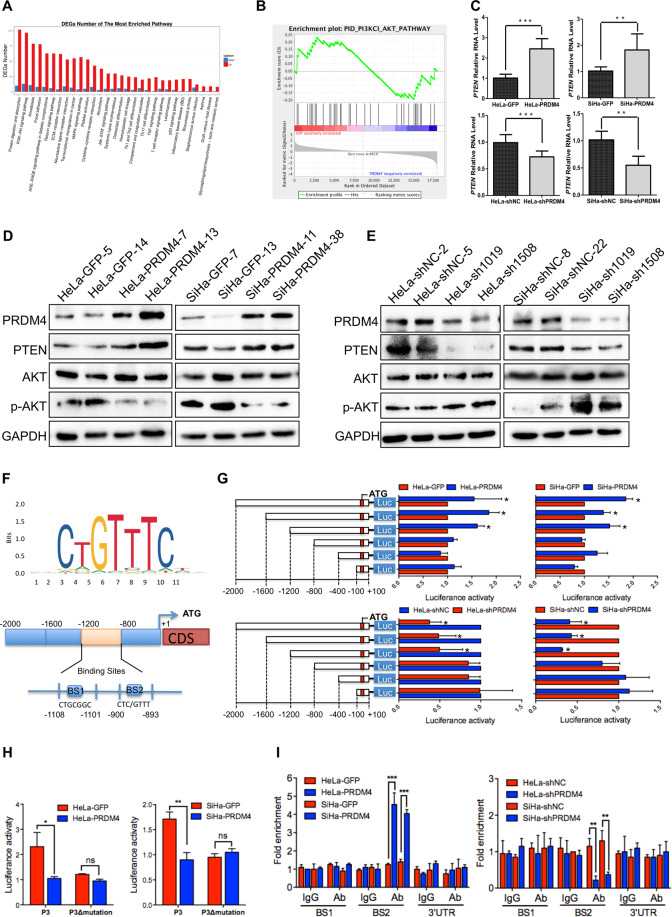
PRDM4 inhibited the activity of the PI3K/AKT pathway by transactivating *PTEN*. **A**, **B** Bioinformatics analysis of RNA-seq was performed to confirm that the PI3K/AKT pathway and target genes were significantly enriched in HeLa-PRDM4 cells. **C** The mRNA level of *PTEN* was detected by real-time PCR. **D**, **E** The expression levels of AKT, p-AKT, and PTEN were detected by Western blots. **F** The PRDM4 DNA-binding motif containing TC/AATTA was predicted by the JASPAR database (http://jaspar.genereg.net/), and the diagram of the *PTEN* promoter containing the possible cis-acting elements bound by PRDM4 is also shown. **G** Luciferase activity of *PTEN* promoter deletions relative to Renilla activity was detected in the PRDM4-overexpressing or PRDM4-silenced cells and the controls. **H** The luciferase activity of the *PTEN* promoter P3 mutations was detected in the PRDM4-overexpressing HeLa and SiHa cells. **I** The qChIP assay results are shown for the PRDM4-overexpressing and PRDM4-silenced HeLa and SiHa cells. Statistical significance is denoted by the symbols *p < 0.05, **p < 0.01), ***p < 0.001, while ns indicates no significant difference.

Bioinformatics analyses were conducted with RNA-Seq and gene set enrichment analysis and indicated that the PI3K/AKT pathway was significantly enriched in the HeLa-PRDM4 cells (Fig. 5A, B, FDR < 0.05). An inhibitor of the PI3K/AKT pathway, *PTEN*, was shown by real-time PCR to be increased in the PRDM4-overexpressing cells and decreased in the PRDM4-silenced cells, as revealed by our RNA-Seq results (Fig. 5C, *p* < 0.05). Then, Western blots confirmed that the protein level of PTEN was increased in both the PRDM4-overexpressing HeLa and SiHa cells, with a decrease in p-AKT (Thr308) (Fig. 5D). In contrast, PRDM4 silencing in HeLa and SiHa cells resulted in downregulation of PTEN expression, with an increase in p-AKT expression (Fig. 5E). These results indicated that PRDM4 might inhibit cell proliferation by inhibiting the PI3K/AKT signaling pathway.

PRDM4 should bind to the DNA motif of TC/AATTA (http://jaspar.genereg.net/), which is located in the *PTEN* promoter, as predicted by an integrated web tool for transcription factor binding sites, LASAGNA-Search 2.0 (https://biogrid-lasagna.engr.uconn.edu/, Fig. 5F). To test whether PRDM4 transcriptionally regulated the expression of PTEN, we constructed *PTEN* promoter regions (−2000 to +100 bp) and promoter deletions. The luciferase activity of P3 (−1200 to +100 bp) in the PRDM4-overexpressing cells was increased, whereas PRDM4 silencing reduced P3 luciferase activity (Fig. 5G). Then, we generated a mutation in the P3 promoter reporter without the P3Δmutation. Notably, mutation of the binding sites abolished the PRDM4-mediated promotion of promoter activity (*p* < 0.05, Fig. 5H). These data suggested that PRDM4 transactivated PTEN expression by binding to the −1200 to −800 bp region of the *PTEN* promoter in HeLa and SiHa cells.

Furthermore, a quantitative chromatin immunoprecipitation (qChIP) assay was performed to determine whether the PRDM4 protein directly binds to the −1200 to −800 bp region of the *PTEN* promoter. Two pairs of primers were designed to amplify the two sites, binding site 1 and binding site 2, of the −1200 to −800 bp regions (Fig. 5F). BS1 (−1108 ~ − 1101 bp) was a random sequence, and BS2 (−900 to −893 bp) contained the PRDM4-binding motif CTC/GTTT. Significantly more BS2 promoter fragments were amplified by real-time PCR in the HeLa-PRDM4 and SiHa-PRDM4 cells than in the control cells after immunoprecipitation with a PRDM4 antibody (Fig. 5I, *p* < 0.001). However, there was no significant difference in the amplification of the BS1 promoter fragments. The amplification of any of the two binding site promoter fragments was performed as controls immunoprecipitated by the IgG antibody. All of these results indicated that PRDM4 increased *PTEN* transcription by binding directly to its promoter at the CTGTTT binding sites.

### PTEN silencing or the PTEN inhibitor SP1670 rescued the cell proliferation induced by PRDM4 overexpression in cervical cancer

**Fig. 6:**
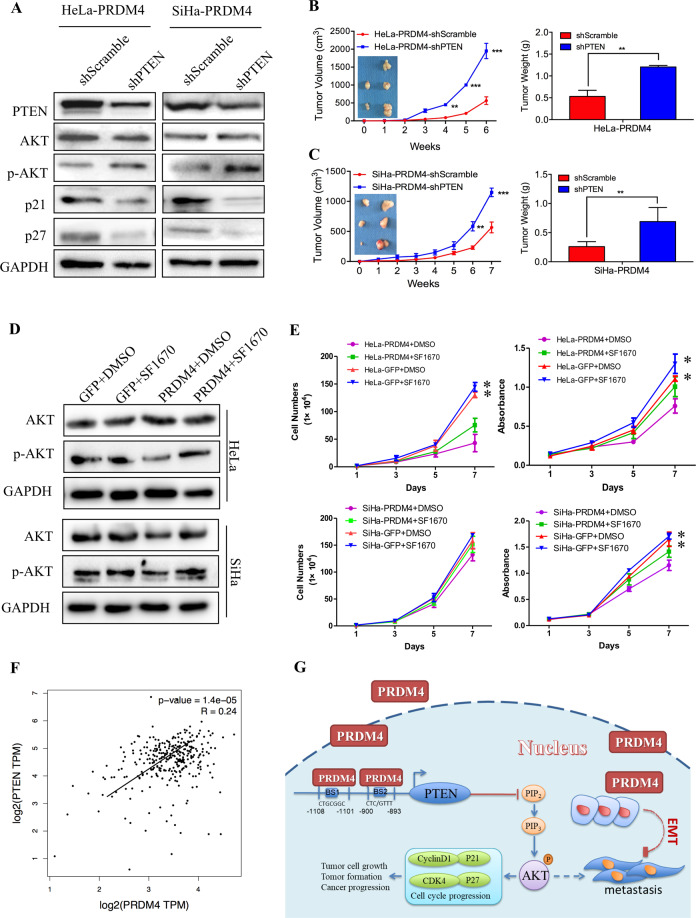
Silencing the expression of PTEN rescued the cell growth potential induced by PRDM4 overexpression. **A** The expression levels of PTEN, AKT, p-AKT, p21, and p27 were detected by Western blots in the PTEN-silenced HeLa-PRDM4 and SiHa-PRDM4 cells. **B**, **C** PTEN was silenced by specific shRNA targeting the *PTEN* CDS region in the PRDM4-overexpressing HeLa and SiHa cells, and the associated tumor weights and growth curves are shown. **D** The expression levels of AKT and p-AKT were detected in the HeLa-PRDM4 and SiHa-PRDM4 cells treated with the PTEN inhibitor SF1670. **E** The cell proliferation and cell vitality were assessed in the HeLa-PRDM4 and SiHa-PRDM4 cells treated with the PTEN inhibitor SF1670. **F** The correlation between the PRDM4 and PTEN mRNA levels in cervical cancer was analyzed using the TCGA database (*R* = 0.24, *p* < 0.05). **G** A proposed model of the PRDM4-mediated enhancement of PTEN inactivation of the PI3K/AKT pathway in cervical cancer progression. The data are presented as the mean ± SD of experiments in triplicate. Statistical significance is denoted by the symbols *p < 0.05, **p < 0.01, ***p < 0.001.

To further confirm that PRDM4 inactivated the PI3K/AKT signaling pathway by transactivating *PTEN*, we stably transfected the shPTEN and control plasmids into HeLa-PRDM4 and SiHa-PRDM4 cells, respectively. The p-AKT (Thr308) protein levels were much higher in the cells transfected with shPTEN than in the cells transfected with the control plasmids, with inhibition of p27 and p21 protein expression (Fig. 6A). In addition, the tumor formation inhibited by PRDM4 overexpression was reversed when PTEN was silenced (*p* < 0.05, Fig. 6B, C). Furthermore, when cells were treated with a PTEN-specific inhibitor, SP1670, for 24 h at a concentration of 2 µmol/ml, the levels of p-AKT were increased compared with those in the DMSO control cells (Fig. 6D). Additionally, the HeLa-PRDM4 and SiHa-PRDM4 cells treated with SP1670 showed much higher cell proliferation and viability (*p* < 0.05, Fig. 6E). Additionally, the TCGA database showed that the mRNA level of *PRDM4* in cervical cancer tissues was significantly positively related to the *PTEN* mRNA level, with an R of 0.25 (*p* < 0.01, Fig. 6F).

All of these results further confirmed that PRDM4 inhibited the proliferation and tumorigenicity of cervical cancer cells by suppressing the PI3K/AKT signaling pathway via the transactivation of *PTEN* (Fig. 6G).

## Discussion

The malignant transformation of normal cervical epithelium is caused by high-risk HPV infection combined with genetic and epigenetic abnormalities. Here, viral DNA was detected in cervical cancer patients using the universal primers GP5+/6+, and the PRDM4 IRS was 3.86 ± 0.82 in HPV-positive and 4.13 ± 1.31 in HPV-negative samples, indicating that there was no significant difference between PRDM4 expression and HPV infection (Supplementary Table 1). Additionally, PRDM4 showed a certain percentage of positive samples in SCC. These results indicated that PRDM4 is one of the cancer-related factors involved in cell proliferation and malignant biological processes. Moreover, the function of PRDM4 might be replaced by other PRDM factors.

In the cancer process, most PRDM family proteins, including PRDM4, are catalytically inactive. However, it has been reported that PRDM4 interacts with the WW domains of YAP to mediate the expression of ITGB2 and other YAP target genes, inducing cell invasion and tumorigenesis in prostate cancer [21]. A previous study showed that *PRDM4* maps to a tumor suppressor locus on human chromosome 12q23-q24.1 [20]. Additionally, as it is a critical mediator for neural stem cells, PRDM4 recruits protein arginine methyltransferase 5 to mediate histone arginine methylation and control cell proliferation and differentiation [22]. Additionally, *PRDM4*, as the target gene of the PI3Kα-AKT1 signaling pathway, has been shown to increase energy expenditure, inhibit weight gain and improve insulin resistance [23]. Here, we found that PRDM4 inhibited cell proliferation and tumor formation in cervical cancer by blocking the cell cycle transition from G0/G1 to S phase by regulating cell cycle checkpoint factors, including p21, p27, Cyclin D1, Cyclin E, and CDK4. As a key factor, CDK1 generally cooperates with Cyclin B and p21 to influence the G2/M phase and as the target of the PUM1 impaired trophoblast invasion by negatively regulating the expression of the lncRNA HOTAIR [24, 25]. Here, the proportion of PRDM4-modified cells in G2/M phase was not changed. Therefore, we confirmed that PRDM4 inhibited the cell cycle transition of G0/G1 to S phase by regulating the expression of CDK4/Cyclin D1/p27/p21. Cyclin D1, as a cell proliferation promoter, has been confirmed in different types of cancers [26]. Additionally, the strong and widespread roles of p53 and p21 in regulating cell invasion and proliferation in several types of cancers and trophoblasts have been confirmed, and cervical cancer cells modified by PRDM4 show similar biological behavior [27].

Recently, tumor stage, tumor size, and lymph nodes (LNs) were found to be significantly correlated with both *PTEN* promoter hypermethylation and *PTEN* loss in cervical cancer [28, 29] and showed a significant increase with tumor progression [30]. Regardless, *PTEN* deletion or mutation is an important event that induces the activation and inactivation of signaling pathways in carcinogenesis, including the PI3K/AKT pathway [31]. The upregulation of PTEN expression can inactivate the PI3K/AKT signaling pathway and block human tumor progression [32]. Here, PRDM4 was shown to inhibit cell proliferation and tumorigenesis by decreasing the activity of the PI3K/AKT pathway by transactivating PTEN in cervical cancer. As reported previously, the PI3K/AKT pathway is a regulator of cell migration and invasion in both cancer cells and trophoblasts [33]. Here, we also found that PRDM4 could inhibit the migration and invasion of cervical cancer cells via the PI3K/AKT pathway (Supplementary Figs. 3A–D and 4A–E). Additionally, the levels of EMT-related factors were negatively related to PRDM4 expression, as detected by IHC (Supplementary Fig. 5A). Additionally, to determine whether PRDM4 could result in any level of malignant transformation, PRDM4 siRNA was transiently transfected into HEK293T cells. Notably, the PTEN/PI3K/AKT pathway was consistently improved with no significant difference in morphology or the p53 and H-Ras protein levels in the PRDM4-silenced cells (Supplementary Fig. 6A). These results suggested that PRDM4 could increase the progression of cancer cells rather than initiate malignant transformation. There was no evidence that alteration of PRDM4 affected anything beyond cell proliferation in cervical cancer. These results imply that PRDM4 is involved in the proliferation and metastasis of cervical cancer cells by altering target genes by blocking the PTEN/PI3K/AKT pathway. Additionally, whether the mechanism of PRDM4-mediated *PTEN* transcriptional regulation in cervical cancer involves the recruitment of a posttranscriptional modification-related protein that plays a negative regulatory role requires more in-depth research [33, 34].

In conclusion, our study is the first to demonstrate that PRDM4 inactivates the PI3K/AKT signaling pathway by binding to the *PTEN* promoter and transactivating *PTEN*, blocking the cell cycle process by upregulating p21 and p27 expression and downregulating Cyclin D1 and CDK4 expression, ultimately inhibiting the cell proliferation and tumor growth of cervical cancer cells.

## Materials and methods

### Human tissue specimens

All human tissue specimen data were collected from the First Affiliated Hospital of Xi’an Jiaotong University from 2012 to 2017. None of the patients had received chemotherapy, immunotherapy, or radiotherapy before collection. Normal cervical samples were obtained from patients undergoing total hysterectomy due to hysteromyoma. The fresh samples obtained during surgery were immediately frozen in liquid nitrogen for subsequent protein extraction and fixation in formalin for paraffin embedding. The histological classifications and clinical stage were performed in accordance with the International Federation of Gynecology and Obstetrics classification system. The institutional review board, the Ethics Committee of Medical School of Xi’an Jiaotong University in Shaanxi, China, approved the population study, and all the patients provided informed consent prior to specimen collection. All the data from human tissue specimens are supplied in the Supplementary materials and methods.

### Immunohistochemistry and immunocytochemistry

The immunohistochemical staining procedure was performed as previously described [12]. The antibodies used were as follows: anti-PRDM4 (1:200 dilution, #ab126939, Abcam, Cambridge, MA, USA), anti-Ki67 (1:400 dilution, #sc-23900, Santa Cruz, Biotechnology Inc., Santa Cruz, California, USA), anti-E-cadherin (1:150 dilution, #sc-8426, Santa Cruz, CA, USA), anti-vimentin (1:200 dilution, #sc-6260, Santa Cruz, CA, USA), and anti-β-catenin (1:150 dilution, # sc-7963, Santa Cruz, CA, USA).

### Western blot analysis

The Western blot procedure was performed as previously described [35]. The antibodies used are shown in Supplementary Table 2. The signals of the antigen–antibody complexes were detected by an enhanced chemiluminescence solution (Millipore, Billerica, MA, USA). The expression level of target proteins was determined by densitometric semiquantitative analysis. Briefly, the abundance of target proteins was densitometrically determined by utilizing a protein imprinting imaging system (Tanon 5200, Shanghai, China) and is expressed as relative expression after normalization by GAPDH.

### Reverse transcription-PCR (RT-PCR) and real-time PCR

Primers are shown in Supplementary Table 3. See detailed method in the Supplementary materials and methods.

### Xenograft mouse model

Female mice (BALB/c nude, 4–6 weeks old), purchased from the Shanghai SLAC Laboratory Animal Co., Ltd., were used for the xenograft studies. The animals had free access to food and water and were housed with a 12 h light–dark cycle and a constant temperature under specific pathogen-free conditions. Twelve mice were randomly divided into two groups, with six mice in each group. PRDM4-overexpressing SiHa and HeLa cells (1 × 10^6^), along with the respective control cells, were harvested in the exponential growth phase and injected (100 μl per site) subcutaneously into the dorsum of each mouse. A similar protocol was performed with the PRDM4-silenced SiHa and HeLa cells with a cell count of 1 × 10^5^ per group. The tumors were measured in two dimensions by using manual calipers every 2–3 days. The tumor volume was calculated using the following formula: *V* = 0.5 × length × width^2^. Approximately 6–8 weeks later, the mice were sacrificed; the tumor weight was measured and subjected to histological examination.

For the tumor-free survival curves, mice xenografted with PRDM4-modified cells and control cells were assessed every 3 days by the same investigator. The appearance of the first palpable tumor was recorded. Statistical analysis was performed using Kaplan–Meier survival studies. Significant differences were considered at *p* < 0.05.

### RNA preparation and transcriptome sequencing

The RNA-seq method is shown in the Supplementary materials and methods.

### Quantitative chromatin immunoprecipitation

qChIP assays were performed according to the manufacturer’s protocol for the EZ-Magna ChIP Kit (Millipore, Darmstadt, Germany). Chromatin-protein complexes were immunoprecipitated with 5 μg of anti-PRDM4 antibodies (#ab126939, Abcam, Cambridge, MA, USA) and 20 μl of fully resuspended protein A/G magnetic beads. For the negative control, 1 μg of normal mouse IgG was used. Real-time PCR was performed to amplify the regions of interest or internal negative control regions. Each sample was assayed in triplicate, and the amount of precipitated DNA was calculated as a percentage of the input sample.

### Statistical analysis

Statistical analysis was performed with SPSS 18.0 software (SPSS, Inc., Chicago, IL, USA). Measurement data were analyzed with the mean ± standard deviation; the two-tailed *χ*^2^ test or Fisher’s exact test was used to determine the significance of the differences between the covariates. Univariate analysis was analyzed by Student’s *t* test (two-tailed) and the Mann–Whitney *U* test. For comparisons among groups, the *χ*^2^ test or one-way ANOVA was performed. A *p* value <0.05 was considered statistically significant.

### Supplementary methods

Additional methodological details are available in the Supplementary materials and methods.

## Supplementary information

Supplementary

Supplementary material and method
