# Supplementary material for: PRDM4 inhibits cell proliferation and tumorigenesis by inactivating the PI3K/AKT signaling pathway through targeting of PTEN in cervical carcinoma
Source: Oncogene. 2021 Apr 12;40(18):3318–30. doi: 10.1038/s41388-021-01765-x (PMC8102194; doi:10.1038/s41388-021-01765-x)
Supplement: Supplementary file 1 — Supplementary [file 41388_2021_1765_MOESM1_ESM.pdf]

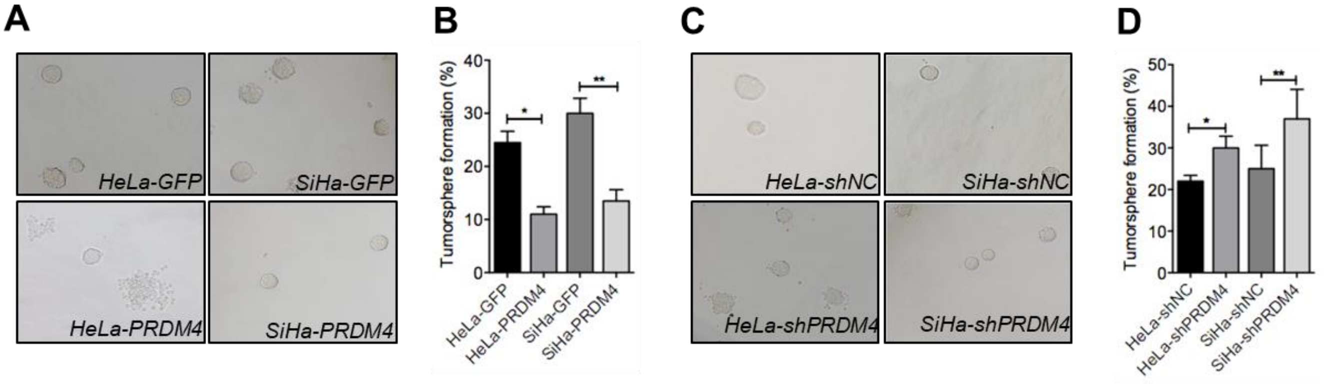

Supplementary Figure 1. PRDM4 inhibited the tumorsphere formation *in vitro*

(A and B) Representative photos of the tumorspheres and the percentage of tumorsphere formation are shown in the PRDM4-overexpressing HeLa and SiHa cells and the control cells. (C and D) Representative photos of the tumorspheres and the percentage of tumorsphere formation are shown in the PRDM4-silenced HeLa and SiHa cells and the control cells. Scale bar, 100 μm. Number of migratory or invasion cells was shown as mean ± SD from three independent experiments using triplicate measurements and statistically analyzed with Student's *t*-test in each experiment. *p < 0.05, **p < 0.01.

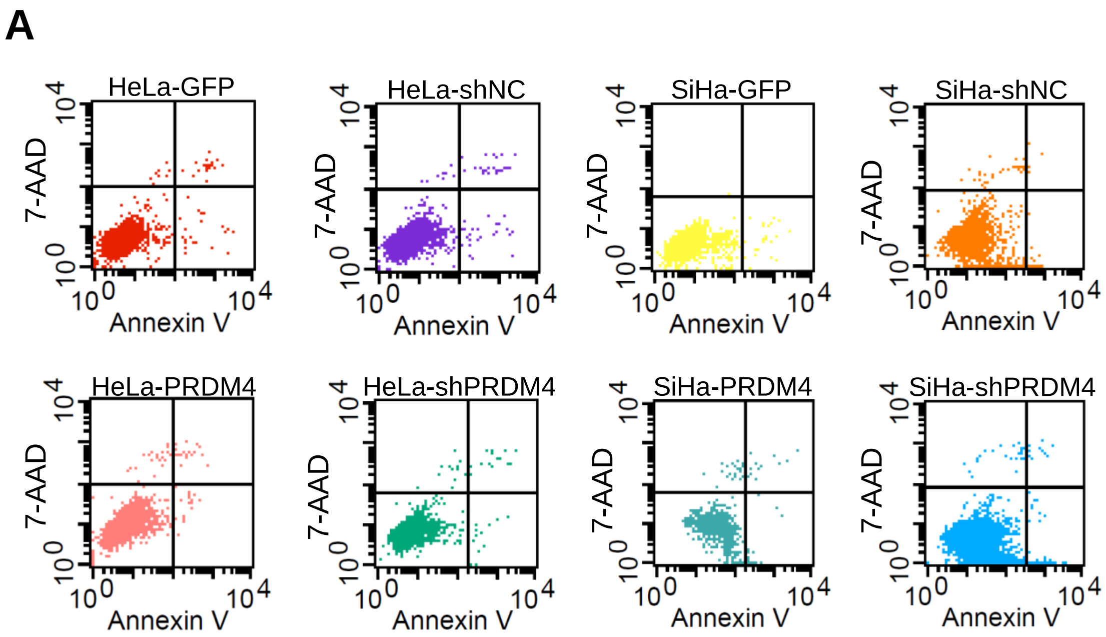

Supplementary Figure 2. PRDM4 had no effect on apoptosis of cervical cancer cells.

PRDM4 modifed SiHa and HeLa cells were harvested on day 3 and stained with APC-Annexin V and 7-AAD Detection kit (BD, United States) according to the manufacturer's instructions. Apoptosis was detected with flow cytometer.

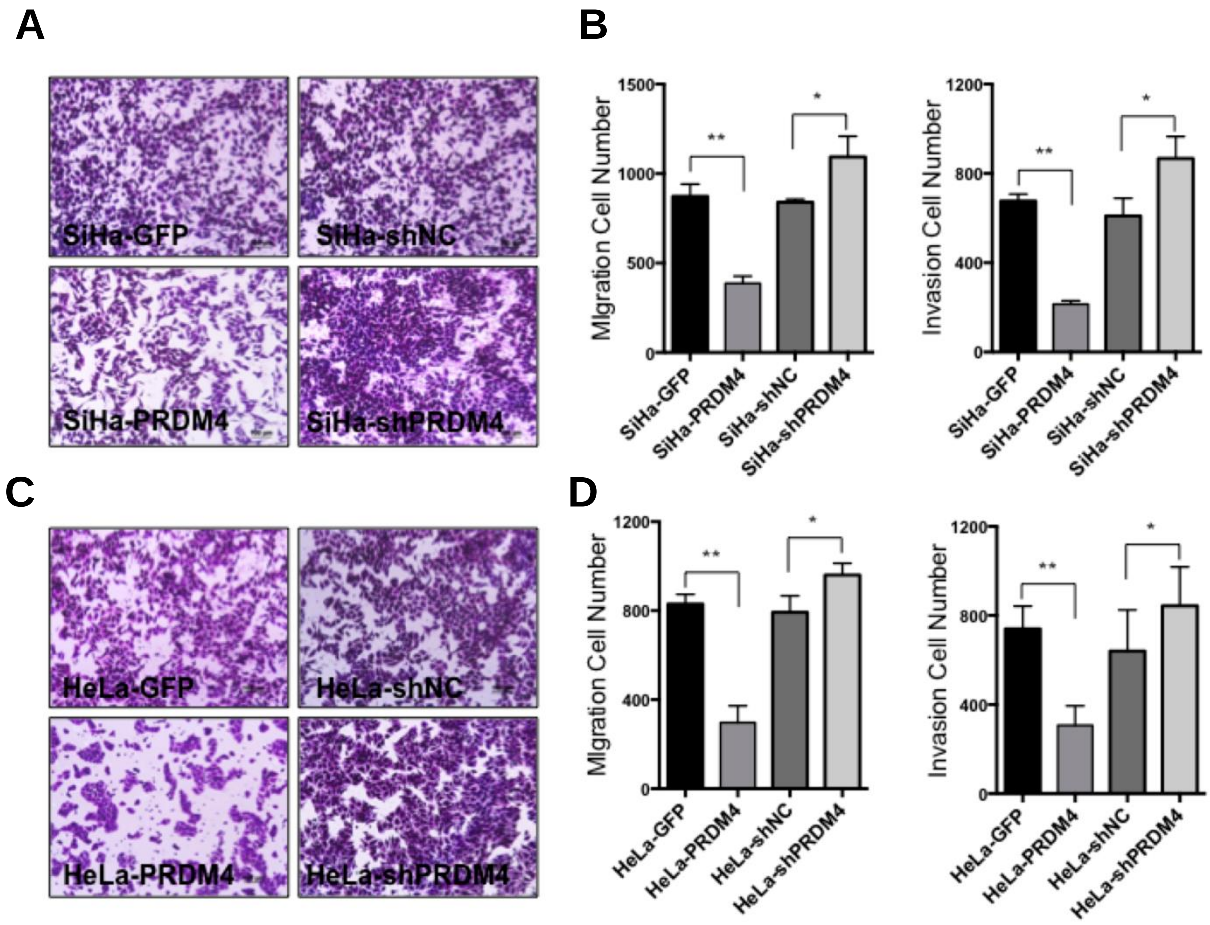

Supplementary Figure 3. PRDM4 blocked migration and invasion ability of SiHa and HeLa cells *in vitro*.

(A and B) The migratory potential of PRDM4-overexpressing or silencing SiHa cells and the respective controls was analyzed by the transwell cell migration and invasion assay. (C and D) The migratory potential of PRDM4-overexpressing or silencing HeLa cells and the respective controls was analyzed by the transwell assay. Scale bar, 100 μm. Number of migratory or invasion cells was shown as mean ± SD from three independent experiments using triplicate measurements and statistically analyzed with Student's *t*-test in each experiment. $*p < 0.05$, $**p < 0.01$

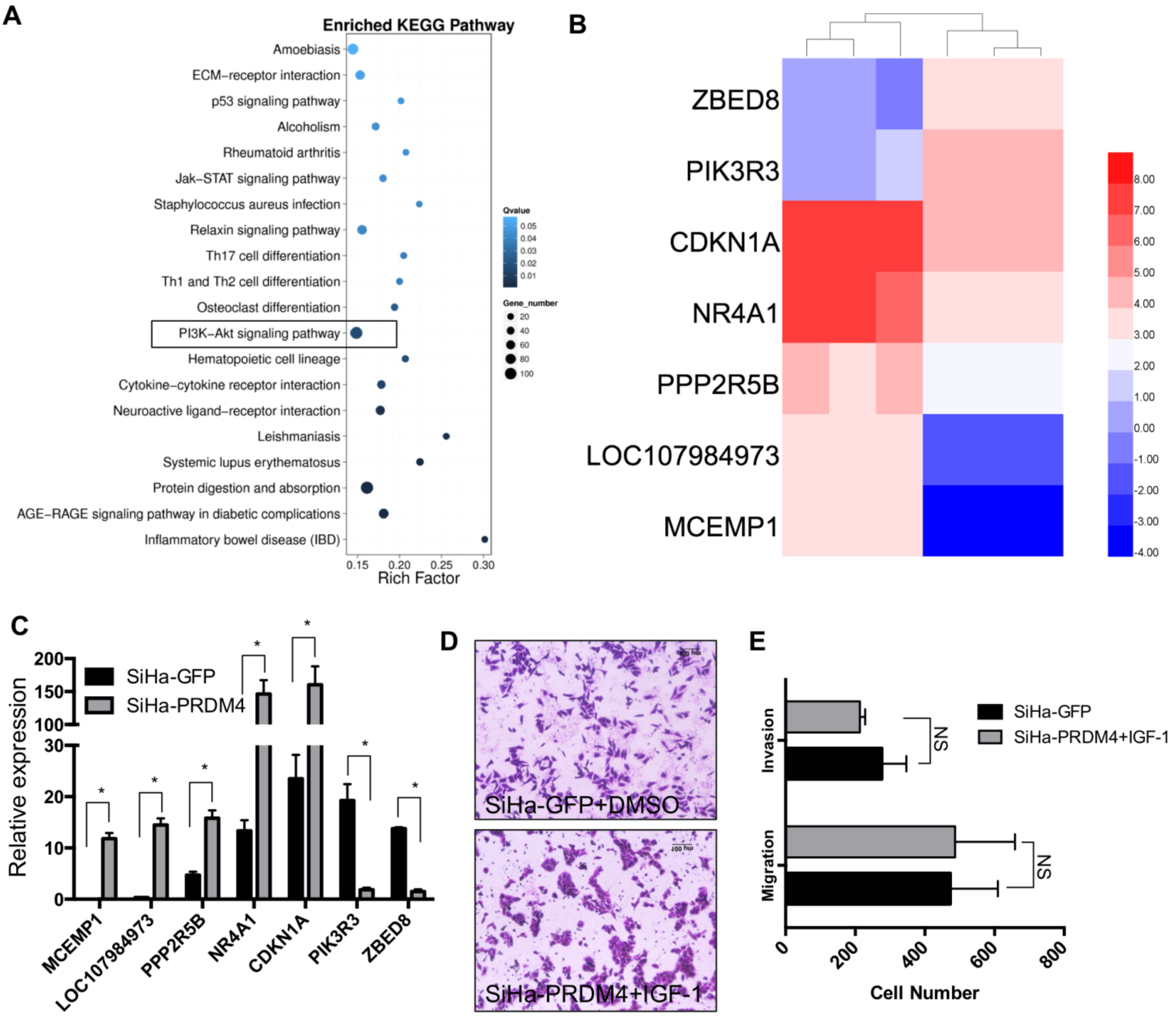

Supplementary Figure 4. PRDM4 downregulated the migration and invasion related genes by inactivated PI3K/AKT pathway

(A) Bioinformatics KEGG pathway analysis of RNA-seq was performed to confirm that the PI3K/AKT pathway was significantly enriched in cells with high expression of PRDM4. (B) Heatmap of all differentially expressed migration and invasion related genes regulated by PI3K/AKT pathway. (C) The mRNA levels of genes in SiHa-PRDM4 and SiHa-GFP cells detected by Real-time PCR analysis. (D) The migration and invasion potential was valued in SiHa-PRDM4 cells treated with the PI3K/AKT agonist. Scale bar, 50 μm. Data represent mean ± SD of three independent experiments and statistical analyzed with Student's *t*-test. *$p < 0.05$.

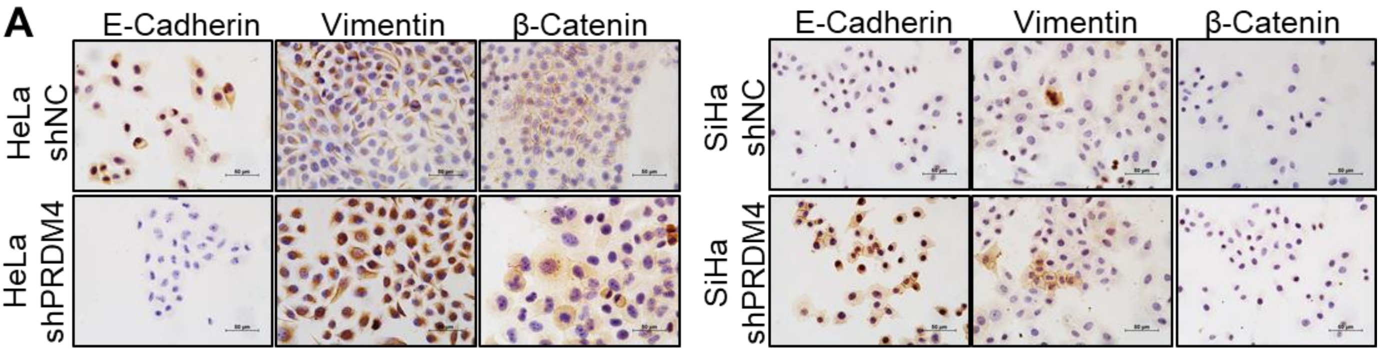

Supplementary Figure 5. Silencing PRDM4 facilitates the epithelial–mesenchymal transition (EMT) related proteins.

(A) The expression level of E-Cadherin, β-Catenin and Vimentin in PRDM4-silencing HeLa or SiHa cells and the respective control cells were detected by Immunocytochemistry. Scale bar, 50 μm.

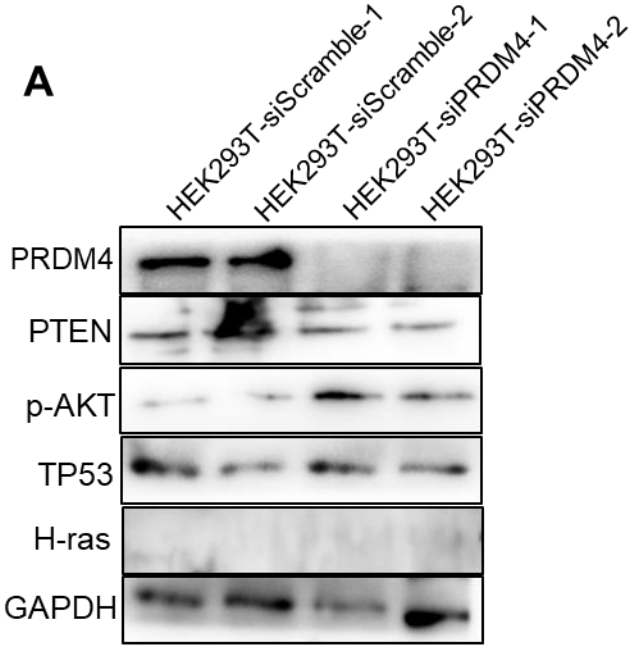

Supplementary Figure 6. Silence of PRDM4 in non-tumor cells HEK293T cells

(A) The expression of oncogene p53, H-Ras, PTEN and p-AKT were detected in PRDM4 silencing HEK293T cells by Western blot.

Supplementary Table 1. PRDM4 expression in cervical cancer patients with different HPV status

HPV status	n	PRDM4 IRS	p-value
positive group	19	3.86±0.82	0.32
negative group	3	4.13±1.31	

Note: For comparison, the *t*-test was performed. A p value <0.05 was considered statistically significant.

Supplementary Table 2. Antibody used in western blot

antibody	dilution	Lot#
anti-PRDM4	1:1000	#ab126939, Abcam, Cambridge, USA
anti-GAPDH	1:1000	#sc-47724, Santa Cruz, USA
anti-p27	1:1000	#sc-53936, Santa Cruz, USA
anti-p21	1:500	#sc-56335, Santa Cruz, USA
anti-AKT	1:500	#sc-5298, Santa Cruz, USA
anti-p-AKT	1:1000	#13038, p-AKT (Thr308), Cell Signaling Technology, USA
anti-CyclinD1	1:500	#sc-8396, Santa Cruz, USA
anti-CyclinE	1:500	#sc-247, Santa Cruz, USA
anti-CDK4	1:500	#sc-23896, Santa Cruz, USA
anti-H-ras	1:500	#sc-35, Santa Cruz, USA
anti-TP53	1:500	#sc-126, Santa Cruz, USA
HRP conjugated anti-rabbit or anti-mouse IgG	1:10000	#21234 and 32430 Thermo Fisher Scientific, NY, USA

Supplementary Table 3. The primer sequences used for the cell cycle related gene by Real-time PCR

gene name	Sense	Antisense
P53	AAGAAACTGGCGGAATGGC	CCAAGAACCACCCCTGAGTC
P27	CTGCCCTCCCCAGTCTCTCT	CAAGCACCTCGGATTTT
P21	GCAGACCAGCATGACAGATTTC	CGGATTAGGGCTTCCTCTTG
CDK4	ATGTTGTCCGGCTGATGGA	CACCAGGGTTACCTTGATCTCC
CyclinD1	AAACAGATCATCCGCAAACAC	GTTGGGGCTCCTCAGGTTC
PTEN	GCTTGTCAATCCCTTCTACG	CAGCCAACCATCTTGTCTAACT
GAPDH	GCACCGTCAAGGCTGAGAAC	TGGTGAAGACGCCAGTGGA

Material and methods for the Supplementary data

Apoptosis analysis by FACS

Apoptosis was analyzed *in vitro* using the FACS Annexin V assay kit (BD Biosciences) according to the manufacturer's instructions. Briefly, cells were harvested at 1×10^5 cells per tube, and stained in duplicate with 5 µl of APC-Annexin V conjugate and 5 µl of propidium iodide (10 µg/ml) for 30 min in the dark. Subsequently, the samples were analyzed by a FACS Calibur flow cytometry (Becton Dickinson, Franklin Lakes, NJ, USA). The percent of apoptotic cells is referred to as the apoptotic index.

Migration and invasion assays

Trans-well chambers (Corning, Corning, NY, USA) equipped with 8-µm pore insets were used for the migration and invasion assays. For the migration assay, 8×10^4 PRDM4-overexpressing SiHa or HeLa cells and the respective control cells in serum-free medium were plated on uncoated insets and incubated for 48 h. For the invasion assay, the insets were coated with 70 µL of 1:8-diluted Matrigel (BD Biosciences), and 1×10^5 cells were plated in the serum-free medium described above for an incubation period of 48 h. Quantities of 600 µL of culture medium containing 20% FBS (Invitrogen) were added to the lower chamber. Non-invaded cells were removed, and the cells that were attached to the bottom of the membrane were fixed with 4% paraformaldehyde, stained with 5% crystal violet (Sigma-Aldrich), and counted at 200-fold magnification. The experiment was repeated in three independent experiments.
