# Supplementary material for: PRDM4 inhibits cell proliferation and tumorigenesis by inactivating the PI3K/AKT signaling pathway through targeting of PTEN in cervical carcinoma
Source: Oncogene. 2021 Apr 12;40(18):3318–30. doi: 10.1038/s41388-021-01765-x (PMC8102194; doi:10.1038/s41388-021-01765-x)
Supplement: Supplementary file 2 — Supplementary material and method [file 41388_2021_1765_MOESM2_ESM.pdf]

Supplementary materials and method

Data acquisition

RNA-sequencing, clinical data and survival analyses were acquired using TCGA from cervical cancer tissues (N=304) and normal cervical tissues (N=12). The gene set enrichment analysis (GSEA) software was obtained from the Broad Institute (http://www.broad.mit.edu/gsea). Normalized TCGA expression data or RNA-Seq gene expression data were analyzed using GSEAv4.0. Interrogated signatures were from the MySigDB v7.0 H hallmark gene set database.

Cervical cancer cell lines and cell culture

The human cervical cancer cell lines (HeLa, SiHa, C33A and CaSki) were purchased from the American Type Culture Collection (ATCC; Manassas, VA) and were authenticated by STR profiling. The HeLa, SiHa, and C33A cells were cultured in Dulbecco's modified Eagle's medium (DMEM; Sigma-Aldrich, St Louis, MO, USA); CaSki cells were cultured in RPMI/1640 (Sigma-Aldrich) supplemented with 10% heat-inactivated fetal bovine serum (FBS; HyClone, Waltham, MA, USA). All cell lines were maintained at 37 ° C with 5% CO2.

Reverse transcription-PCR (RT-PCR) and Real-time PCR

Total RNA was isolated using TRIzol reagent according to the manufacturer's instructions (Invitrogen, Carlsbad, CA, USA). Briefly, samples were vortexed with chloroform and centrifuged for 10min, thereby yielding three phases. The upper aqueous phase was precipitated with isopropanol and centrifuged for 15min. The RNA pellets were washed with ethanol, air-dried, and then dissolved in nuclease-free water. Total RNA was reverse transcribed into cDNA using a PrimeScript RT Reagent Kit (TaKaRa, Tokyo, Japan). PrimeSTAR HS DNA Polymerase (TaKaRa) was used for PCR amplification, and SYBR Green Master Mix (TaKaRa) was used for Real-time PCR. The relative expression was calculated by the comparative $2^{-\Delta\Delta Ct}$ method

Cell proliferation and cell cycle assays

For the cell counting assay, cells (1×104) were seeded in 6-well plates for 7 days and counted every 2 days. For the MTT assay, cells (1000) in 96-well plates were treated with 3-(4, 5-dimethylthiazolyl)-2, 5-diphenyl tetrazolium bromide (Sigma-Aldrich) for 7 days and the OD values were measured at 490nm. For the tumorsphere formation assay, 200 cells were incubated in 24-well plates with FBS-free medium, and the visible tumorspheres were photographed and counted. For the cell cycle analysis, cells were harvested and fixed with 70% cold ethanol and stained with solutions containing propidium iodide and DNase-free RNase A (Sigma-Aldrich) for 30min. samples were run on a FACSCalibur flow cytometer (BD Biosciences) using CellQuest software. The results were analyzed with FlowJo_V10 cytometer software.

Vector construction and transfection

Human full-length PRDM4 (NM_012406) cDNA was amplified by RT-PCR with the primer sequences as follows: PRDM4-F: 5-′CCGGTCGACGAAAACATGCATCACAGGATG-3′; PRDM4-R: 5-′CGCGGATCCGTTATTTATGTGCAGAAAGA-3′. The PRDM4 cDNA fragment was subsequently cloned into the SalI and BamHI (TaKaRa, Tokyo, Japan) sites of an internal ribosome entry site vector, pIRES2-AcGFP1-Neo (Clontech, Mountain View, CA). Transfection was performed using Lipofectamine 2000 reagent (Invitrogen, Carlsbad, CA, USA) according to the manufacturer’s instructions and then maintained as monoclonal cells treated with G418 (1g/L). Oligonucleotides corresponding to PRDM4- and PTEN-specific small hairpin RNA (shRNA) sequences were purchased from the Shanghai GenePharma Co., Ltd.

Vector construction and transfection

Human full-length PRDM4 (NM_012406) cDNA was amplified by RT-PCR with the primer sequences as follows: PRDM4-F: 5-′CCGGTCGACGAAAACATGCATCACAGGATG-3′; PRDM4-R: 5-′CGCGGATCCGTTATTTATGTGCAGAAAGA-3′. The PRDM4 cDNA fragment was subsequently cloned into the SalI and BamHI (TaKaRa, Tokyo, Japan) sites of an internal ribosome entry site vector, pIRES2-AcGFP1-Neo (Clontech, Mountain View, CA). Transfection was performed using Lipofectamine 2000 reagent (Invitrogen, Carlsbad, CA, USA) according to the manufacturer’s instructions and then maintained as monoclonal cells treated with G418 (1g/L). Oligonucleotides corresponding to PRDM4- and PTEN-specific small hairpin RNA (shRNA) sequences were purchased from the Shanghai GenePharma Co., Ltd.

Dual-luciferase reporter assay

Fragments of the *PTEN* promoter (predicted from position -2000 bp to +100 bp) were cloned into the pGL3-Basic Vector (Promega, Madison, WI, USA) to generate promoter reporter constructs. All constructs were verified by sequencing. The cells were seeded in 24-well plates and transiently transfected with plasmids containing firefly luciferase reporters and recombinant promoter reporter constructs. The luciferase activity was measured after incubation for 48 h using a dual-luciferase assay kit (Promega). All experiments were performed as three independent experiments. The transfection efficiency was normalized with *Renilla* luciferase activity. The specific promoter activity was presented as the change in the experimental group versus the control group. The specific activity was shown as the fold change of the experimental group versus the control group.

RNA preparation and transcriptome sequencing

RNA was extracted using TRIzol reagent. Samples were measured using the BGISEQ-500 platform, and the average output of each sample was 11.03GB. The experiment analysis used the NOISeq method for the identification of differentially expressed genes (DEGs) based on a log2-fold change>1 and a probability≥0.80. According to the results of GO annotation and official classification, we classified the DEGs and performed the analysis by using the Phyper function in R software. The FDR was then corrected for p value, and the function of FDR≤ 0.01was usually seen as significant enrichment.
